# Using reflexivity to enhance in-depth interviewing skills for the clinician researcher

**DOI:** 10.1186/1471-2288-8-73

**Published:** 2008-11-09

**Authors:** Ruth McNair, Angela Taft, Kelsey Hegarty

**Affiliations:** 1 The Department of General Practice, University of Melbourne, 200 Berkeley St, Carlton, 3053, Victoria, Australia; 2 Mother and Child Health Research, La Trobe University, 324-328 Little Lonsdale St, Melbourne, 3000, Victoria, Australia

## Abstract

**Background:**

Primary health care clinicians are being encouraged to undertake qualitative research, however the in-depth interviewing skills required are not as straightforward as might be first supposed. While there are benefits and certain skills that clinicians can bring to interview-based research, there are important new skills to develop. To date there has been neither discussion about these new skills, nor any preparatory guidelines for clinicians entering into interview-based research in the qualitative research literature. In the absence of formal guidelines, we suggest the use of reflexivity throughout the interview process as a means to become more accomplished in this area. We present our own experiences as a novice general practitioner (GP) researcher undertaking a PhD study and her experienced supervisors. The PhD study used critical phenomenology through in-depth interviews to understand the experience of the patient-doctor relationship between same-sex attracted women and their usual GP in Australia.

**Results:**

We used reflexivity to improve the rigour of the data collection. This enabled improved probing, fewer assumptions, avoidance of premature interpretation, and an accentuated sense of curiosity during interviews. We also enlisted reciprocity between interviewer and interviewee as a tool to improve engagement and trust, share interview control, and ultimately improve the depth of the interview content.

**Conclusion:**

Preparatory recommendations for novice clinician research interviewers include the importance of recognising the multiple identities that they bring to the interview. In this setting in particular this involves acknowledging the clinician interviewer as a potential insider in relation to interviewees and negotiating shared understanding to avoid insider assumptions. Other essential requirements are having an experienced research supervisor, arranging pilot interviews that include active feedback on interviewing style from interviewees, and being reflexive during interviews. More formal guidelines for in-depth interviewing skills development are needed.

## Background

### Benefits and pitfalls of clinicians as qualitative researchers

Primary health care clinicians have been encouraged to become involved in research over the past decade [1,2], and funding of PhD scholarships and post-doctoral fellowships for clinicians has made this more possible. Clinician researchers are envisaged as an untapped resource, particularly due to the range of benefits that they can bring to qualitative research from the beginning to the end of the process. These benefits occur at all stages of the research process including the ability to select research questions that are clinically relevant, the choice of practical research settings and access to the clinical field, the addition of tacit clinical knowledge to the analysis, the will to report research findings in a clinically applicable way, and an ongoing commitment to the researched population [3]. Further, clinicians interviewing other clinicians are insider researchers in that they share at least some understanding of the clinical environment and may share some values [4]. Clinician researchers can therefore enhance qualitative health research by being able to provide a depth of understanding to the meanings practitioners bring to the health care environment [5]. A further value is that a clinician may be placed in a  position of greater trust by consumer and clinician participants by virtue of their status and experience in the field, therefore encouraging research participation and the exploration of sensitive issues [6].

The potential pitfalls of being a clinician researcher have been less well recognised. Some of the traps can be drawn from the extensive literature on the so-called "insider-outsider controversy" [[7], p.182] as clinicians can be seen as insiders on a number of levels [8]. The issues include whether an insider is the most appropriate person to research their own community or domain. Ethical considerations that are particularly pertinent include the risk of coercion of participants [4], and the potential for the blurring of role boundaries between researcher and participant [9]. The rigour of the project could also be compromised if clinician researchers fail to recognise their "shared conceptual blindness" [[10], p.288] with clinician participants during the interview and analysis phases or fail to fully report compromising findings [4].

### Comparisons between clinical and in-depth research interviewing

In-depth interviewing in primary health care research is a popular method to understand the health care experience and the patient-clinician relationship. It has been defined as "a conversation between researcher and informant focusing on the informant's perception of self, life and experience, expressed in his or her own words" [[7], p.61]. Clinicians commonly bring qualities such as a genuine interest in others and a respect for the stories told (not limited to clinicians), but also have been trained in skills involving observation and non-verbal communication, and a range of questioning and responding techniques such as open-ended questions [7]. These pre-existing clinical skills are an excellent starting point for the researcher. It has been argued that the clinical interview is more complex than the research interview due to competing agendas, the propensity for disagreement and difficulty in achieving "a shared perception of the facts" [[7], p.133], whereas the aims of the research interview may often be "mutually congruent" [[7]. p.132]. Interviewing skills do vary across different clinical disciplines, with the brief and largely deductive medical interview being more dissimilar to the in-depth interview than the counselling interview, which tends to be longer and narrative based. The patient-centred clinical method, which is now favoured by many general practitioners, also fosters interviews that are more conversational and empathic than traditional medical interviews, making these skills more transferable to a research interview [11].

### Using reflexivity to overcome pitfalls

Reflexivity has been defined as "an effort to reflect on how the researcher is located in a particular social, political, cultural and linguistic context" [[12], p.179]. This is a central point of divergence for clinicians who are traditionally trained to remove all but remnants of the self from the consultation in order to focus exclusively on the patient. Focusing on oneself as the interviewer can highlight our assumptions and values that may be subconsciously driving the interview. Reflexivity has been recommended as a means of ensuring that not only the data gathering, but also interpretation of the findings is qualified by this knowledge [13]. The use of reciprocity in research interviews is one reflexive tool. Reciprocity is a feminist-inspired reflexive method in which the researcher shares feelings and experiences with the participants [14]. We will present some of the ways in which we used reflexivity throughout the interview process as a means to become more accomplished in this area.

The discussion that we present arose from the realisation that the novice GP researcher (first author of this paper, RM) was inadequately prepared for her role as an in-depth interviewer in her PhD study, despite being an experienced clinician. We found that there has been little discussion in the qualitative research literature about the additional mindfulness that clinicians need for effective in-depth interviewing and there are no preparatory guidelines [15]. We therefore felt it was useful to present our process of reflection in order to initiate a discussion about what might be included in future guidelines. The PhD study in question used critical phenomenology and in-depth individual interviews with 60 participants (same sex attracted women and their GPs) to understand the experience of the patient-doctor relationship and the place of disclosure of sexual orientation in the consultation. The University of Melbourne Human Research Ethics Committee approved the PhD project. All participants selected their own pseudonym, which have been used within all quotations in this paper. Critical and reflexive feedback from the research team responding to early interview transcripts, and subsequent improved reflexivity of the GP researcher during interviews led to a series of early lessons and subsequent improvements for the novice GP researcher.

## Discussion

Contrary to the assertion that research interviews are less complex, for the novice GP researcher conducting the presented research they presented significant challenges, particularly the move from a largely deductive to a more inductive approach. Method problems such as question sequences that were inflexible, and unsuitable questioning techniques [16] were unconsciously present in the early pilot interviews, and increasingly became evident to the GP researcher during interviews and transcribing. Many of the areas needing improvement that we will discuss will be familiar to any researcher, whether clinician or not, however we suggest they are areas that the novice clinician researcher needs to be particularly aware of. Reading pilot interview transcripts by the two supervisors highlighted these various pitfalls and allowed for a discussion of better and more open approaches. We have summarised the common pitfalls and suggested solution in Table 1, and also present them in detail with examples from our research.

**Table 1:** Summarising pitfalls and recommendations

**Common pitfalls during interviews**	**Techniques to overcome inadequacies**
Insider researcher assumptions and shared conceptual blindness with participants. Clinician discipline about time, which can lead to:	Reflexivity to understand possible effects of the interviewer's clinical background on control, assumptions, and interpretation of findings.
• excessive control of the interview	Reciprocity including willingness to share personal details to enhance the interview.
• inappropriate summary interpretation or paraphrasing	
• inadequate probing for feelings and meanings	

### Control of the interview

The necessity of working under strict time constraints in the clinical setting generates directive skills that focus the primary care interview towards a rapid conclusion. These include interpretive comments, probes containing assumptions, paraphrases and summarising statements to indicate closure and a high level of control of interview content, despite the best attempts at patient-centredness. The novice researcher using these skills can tend to follow the interview schedule rather too strictly, preventing participants from following their own train of thought as seen in the following example, which occurred early in the second pilot interview with a GP using the pseudonym 'Lith':

*Interviewer: Tell me about the range of patients you see.*

Lith: Well, maybe 80% of my patients would be gay, either male or female. (...) Most of the time they're very open about their sexuality. I suppose they feel safe in our clinic so it's not an issue.

*Interviewer: Have you worked elsewhere before this practice?*

Lith: Yes, I worked at the [Clinic in a rural town].

*Interviewer: What was the range of patients there?*

Lith: Oh well that obviously would be... I can't recall ever seeing anyone ...any lesbian or gay people who are out. Not a single one actually in my six months there.

*Interviewer: Quite a contrast.*

Lith: That's right. I mean there's no reason to ask them about their sexuality and there's no reason for them to tell me, so I really have no idea.

*Interviewer: I will come back to that (...). So back to just your general style...*

Lith raised disclosure of sexual orientation twice in two clinical contexts. Rather than taking the lead from Lith and asking why his gay patients felt safe and why he thought there was such a difference between the clinics, the GP researcher deferred that discussion, despite disclosure being central to the study.

### Inappropriate interpretation

Using paraphrasing to check interpretations may be very useful in the clinical encounter, but may be inaccurate in an in-depth interview and was a common early error. For example, to be sure that the GP researcher had understood what 'Esther' was saying about her GP's reaction to her disclosure as a lesbian, she paraphrased what Esther had said:

Esther: (...) with my own GP (...), I can't remember how I would have come out. I think I just said 'I'm so and so's partner, who was already a patient, and I think that what made it really good was it was just normalised, no facial surprise or anything. (...)

*Interviewer: So her reaction was pretty positive in fact.*

Esther: Yes.

Esther initially agree, however later went on to clarify that the reaction wasn't positive, but rather was 'neutral and normalising'. A better response would have been to ask an open question such as 'tell me more about that' or probe such as 'how did that feel for you?' or 'how would you further describe her reaction?'. Esther provided feedback following this pilot interview. She felt the interview was too long and some questions were duplicated, indicating her lack of control over the flow and content. She also suggested that participants be provided with some idea of the content before the interview so they could prepare themselves. These were important reflections that we responded to in subsequent interviews.

### Inadequate probing for feelings and meanings

Probing for factual information is a well-honed clinical skill, whereas in-depth probing for more potentially sensitive emotions can be less practised for the clinician, as can be seen in the following extract from an interview with 'Nede', a lesbian woman:

Nede: I wanted to go on Roaccutane for my skin and she [the GP] insisted on a pregnancy test. I said well for a start I'm menopausal and I'm lesbian. She made me have it anyway, which I found really humiliating.

*Interviewer: How did you react?*

Nede: I just went along with it, but I felt quite powerless. To get what I wanted I had to do that.

*Interviewer: How long ago was that?*

The last question was factual and 'safe', which blocked an opportunity to really understand the emotional repercussions of this experience for Nede. A better response would have been to offer an empathic statement, then perhaps a probe such as 'tell me more about how that felt'.

The example from Nede's interview also reflects a collision of multiple identities for this GP researcher. Rather than containing her clinician identity, she was embarrassed and felt compromised by this story, worrying that Nede's GP's behaviour may have reflected on her profession, generating a reluctance to hear more. Arber, a nurse researcher, has described her own fluid identity, which was constantly being defined and re-defined by herself and the participants during interviews [17]. She suggests the need to carefully distance or bracket ones own experience. The GP researcher, during later interviews, having now experienced these personal feelings and disclosing and discussing them with others, was better able to put the clinician identity aside when required. For example, the interviewer's probing response for feelings in Lucy's interview showed some improvement:

Lucy: To be quite truthful, GPs scare me a bit. You know, they live in a world that I don't understand and I feel that it's a different world.

*Interviewer: What scares you about it?*

Lucy: Oh it's just the whole loss of control issue, you know, I'm not in charge. I'm an adviser by trade, I advise people, I don't get advised.

### Assumptions from clinician 'insider' knowledge

Assumed knowledge between GP interviewer and GP participant was problematic at times as it tended to prevent adequate clarification or the revelation of contrary positions:

Imogen: I can think of several [lesbian patients] who are just completely comfortable with where they're at in their life and it's never been something they've raised. (...) not specifically about the coming out experience (...).

*Interviewer: And how would you feel if someone presented with [coming out] issues?*

Imogen: Fine, it would be easy, (...) I think it would be a very useful thing, if they wanted to talk about it I'd be quite comfortable, and I think I'd feel I would be able to understand the sort of issues that might arise for them.

After this statement the interviewer changed direction and didn't probe Imogen's comment. This demonstrates a lack of adequate curiosity, which is an essential pre-requisite for an excellent in-depth interview. Curiosity can be cultivated however as pre-existing assumptions are put aside. So, rather than assuming Imogen knew the 'issues that might arise', or had the same experience as the interviewer, the interviewer could have asked 'what are those issues?' or 'can you think of an example?'.

### Reflexivity and reciprocity as techniques to overcome inadequacies

Reflexivity encouraged the GP researcher to create a much deeper sense of herself as having multiple identities within the research interview, which at times simultaneously involved being both an insider and an outsider with regard to the participant. These multiple identities not only involved being a GP and researcher, but also identifying as lesbian. There are multiple values and beliefs held by different lesbian women so, again, it was important not to assume similarity with the same sex attracted women being interviewed. The more the potential for diverse knowledge and values was reflexively acknowledged, the more the pre-existing assumptions could be overcome.

The GP researcher also began to allow herself to respond to direct personal questions from interviewees at times, realising that reciprocity could enhance rapport. She always informed participants during the interview preamble that she was both a GP and a lesbian woman. Kirsti, a bisexual woman, referred to this knowledge during her interview:

Kirsti: Do you worry about the fact that you are a lesbian doctor and people might be worried about that when you examine them or something? Has that occurred to you?

*Interviewer: It has occurred to me as a boundary issue. In fact I attend lesbian doctor conferences where we all meet together to talk about these things.*

Kirsti: And do you tell your patients that you are lesbian?

*Interviewer: I work in a practice that is for gay and lesbian patients, so I am often asked by patients whether I'm lesbian and if I am asked I tell them. If I am not asked I don't tell them.*

This exchange during the middle of the interview assisted Kirsti to become more open and reveal her own feelings about seeing GPs. Reciprocity also helped to encourage GP participant reflection. For example, the GP researcher had a brief discussion with one GP participant about her own clinic being popular with lesbian patients, and the need that had arisen for lesbian cultural awareness education of the heterosexual GPs there. She used examples such as learning appropriate terminology, understanding the lesbian social environment and local lesbian support groups. This stimulated the GP participant to consider her own needs for cultural understanding, which was a critical turning point of the interview enabling reflection on her need for behaviour change.

## Summary

Clinician researchers make a valuable contribution to qualitative health research, and this research can add important patient and clinician perspectives, such as meaning and interpretation of the clinical encounter in mixed method clinical trials. However, there is a need to recognise that clinician's privileged access to patients and colleagues for research within our clinical fields must be balanced with a responsibility for rigour in data gathering methods. Here we offer some recommendations that might assist clinician researchers to better prepare for the task of in-depth interviewing.

First, adequate supervision of the research process is invaluable, particularly where the supervisor has expertise in the chosen method themselves and is skilled in the social sciences [17]. Supervisory review of early interview transcripts enables the identification of interviewing skills that require modification, such as inappropriate interpretation or inadequate probing, and particularly alerts the interviewer to unchallenged assumptions related to shared 'insider' backgrounds. Conversely, encouragement to reflect on all of the identities the interviewer brings to the interview, and to utilise these identities in positive ways where appropriate, can give a clinician interviewer permission to overcome their reticence to personally engage. Second, piloting early interviews is crucial. When participants have agreed to be involved in a pilot phase, they can be encouraged to provide feedback to the interviewer. This might include feedback on the flow of the interview and their perceived level of autonomy in the whole process, which can greatly assist in exposing a tendency for excessive control by the interviewer. Finally, the disciplined use of researcher reflexivity during and after interviews enables deliberate modification of the interview style.

These preparatory recommendations merely provide a starting point in what we believe needs to be an ongoing discussion. Insufficient preparation can adversely affect the novice clinician researcher, who might be tempted to return to purely clinical work if early forays into research feel inadequate. Conversely, if the researcher is guided to consider the transferability of their clinical skills while using reflexive modification, the research interviews become rewarding and stimulating for the researcher and beneficial for the research. There may also be reciprocal benefit in transferring skills of reflexivity back to the clinical encounter and thus improving patient centred care. This needs to be tested in future research.

## Competing interests

The authors declare that they have no competing interests.

## Authors' contributions

RM and AT conceived the idea for the paper. RM conducted the interviews, transcribed and analysed the text. RM wrote the first draft of the paper, AT and KH contributed improvements to the text, discussion and conclusions.

## Pre-publication history

The pre-publication history for this paper can be accessed here:



## References

[B1] Britten N, Jones R, Murphy E, Stacy R (1995). Qualitative Research Methods in General-Practice and Primary-Care. Family Practice.

[B2] Malterud K (2001). The art and science of clinical knowledge: evidence beyond measures and numbers. Lancet.

[B3] Reed J, Procter S, Reed J, Procter S (1995). Practitioner research in context. Practitioner research in health care.

[B4] McEvoy P (2001). Interviewing colleagues: Addressing the issues of perspective, inquiry and representation. Nurse Researcher.

[B5] Greenhalgh T, Taylor R (1997). How to read a paper – Papers that go beyond numbers (qualitative research). British Medical Journal.

[B6] Richards H, Emslie C (2000). The 'doctor' or the 'girl from the University'? Considering the influence of professional roles on qualitative interviewing. Family Practice.

[B7] Minichiello V, Aroni R, Timewell E, Alexander L (1995). In-depth interviewing: principles, techniques, analysis.

[B8] Miller WL, Crabtree BF, Denzin NK, Lincoln YS (2003). Clinical research. Strategies of qualitative inquiry.

[B9] Dickson-Swift V, James EL, Kippen S, Liamputtong P (2006). Blurring Boundaries in Qualitative Health Research on Sensitive Topics. Qualitative Health Research.

[B10] Chew-Graham CA, May CR, Perry MS (2002). Qualitative research and the problem of judgement: lessons from interviewing fellow professionals. Family Practice.

[B11] Stewart M (1995). Patient-centered medicine: transforming the clinical method.

[B12] Alvesson M (2002). Postmodernism and social research.

[B13] Alvesson M, Sköldberg K (2000). Reflexive methodology: new vistas for qualitative research.

[B14] Reinharz S, Davidman L (1992). Feminist methods in social research.

[B15] Hoddinott P, Pill R (1997). Qualitative research interviewing by general practitioners. A personal view of the opportunities and pitfalls. Family Practice.

[B16] Fontana A, Frey J, Denzin NK, Lincoln YS (2003). The interview. From structured questions to negotiated text. Collecting and interpreting qualitative materials.

[B17] Arber A (2006). Reflexivity: A challenge for the researcher as practitioner?. Journal of Research in Nursing.

